# Heat shock protein 70 protects the lungs from hyperoxic injury in a neonatal rat model of bronchopulmonary dysplasia

**DOI:** 10.1371/journal.pone.0285944

**Published:** 2023-05-18

**Authors:** Cheng-Han Lee, Tzu-Cheng Su, Ming-Sheng Lee, Chien-Sheng Hsu, Rei-Cheng Yang, Jun-Kai Kao

**Affiliations:** 1 Frontier Molecular Medical Research Center in Children, Changhua Christian Children Hospital, Changhua County, Taiwan; 2 Department of Pathology, Changhua Christian Hospital, Changhua, Taiwan; 3 Department of Pediatrics, Kaohsiung Medical University Hospital, Kaohsiung City, Taiwan; 4 School of Medicine, Kaohsiung Medical University, Kaohsiung City, Taiwan; 5 Institute of Biomedical Sciences, National Chung Hsing University, Taichung City, Taiwan; 6 Department of Post-Baccalaureate Medicine, College of Medicine, National Chung Hsing University, Taichung City, Taiwan; Moti Lal Nehru Medical College, INDIA

## Abstract

Hyperoxia plays a significant role in the pathogenesis of lung injury, such as bronchopulmonary dysplasia (BPD), in premature infants or newborns. BPD management aims to minimize further injury, provide an optimal environment to support growth and recovery. In clinic neonatal care, we need a new therapy for BPD. Heat shock protein 70 (Hsp70) inhibit cell apoptosis and promote cell repair allowing cells to survive lethal injury. We hypothesized that Hsp70 could be used to prevent hyperoxia related BPD in the neonatal rat model through its anti-apoptotic and anti-inflammatory effects. In this study, we explored the effect of Hsp70 on hyperoxia-induced lung injury using neonatal rats. Neonatal Wistar rats were delivered naturally at full term of gestation and were then pooled and randomly assigned to several groups to receive heat stimulation (41°C for 20 min) or room temperature conditions. The Hsp70 group received recombinant Hsp70 intraperitoneally (200 μg/kg, daily). All newborn rats were placed under hyperoxic conditions (85% oxygen) for 21 days. Survival rates in both heat-hyperoxia and Hsp70-hyperoxia groups were higher than those in the hyperoxia group (p < 0.05). Both endogenous and exogenous Hsp70 could reduce early apoptosis of alveolar cells under hyperoxia. Additionally, there were less macrophage infiltration in the lung of the Hsp70 groups (p < 0.05). Heat stress, heat shock proteins, and exogenous recombinant Hsp70 significantly increased the survival rate and reduced pathological hyperoxia induced lung injuries in the development of BPD. These results suggest that treating hyperoxia-induced lung injury with Hsp70 may reduce the risk of developing BPD.

## Introduction

Modern medicine and new technologies have significantly improved the survival rate of premature babies. However, newborns (mostly premature) and infants are prone to bronchopulmonary dysplasia (BPD), a chronic lung disease that results from lung damage caused by mechanical ventilation and prolonged oxygen use [1]. The incidence of moderate to severe BPD in premature infants born at 22–27 weeks of gestation is up to 45% [1], and the mortality rate of very low birth weight infants with BPD within the first year after birth is 23–36% [2]. Infants with BPD have a higher incidence of sudden infant death syndrome and are more likely to develop malnutrition, recurrent pulmonary infection, pulmonary hypertension, retarded intellectual development and obstructive pulmonary disease in the future [2–4]. These complications of BPD result in serious social and family burdens.

BPD is currently classified as either “old BPD” or “new BPD”. Old BPD is associated with prominent fibroproliferation and is currently less prevalent. The new BPD is related to the disruption of distal lung growth, with prominent impairment of alveolar and vascular growth [3, 5]. Unlike mechanical ventilator barotrauma in old BPD, hyperoxia toxicity is the major contributor to the development of new BPD due to the immature status of the lung antioxidant systems in premature infants [3, 4, 6–11]. Prolonged hyperoxia causes dysplastic lungs, increased collagen deposition, increased interstitial thickness, and induces the recruitment of inflammatory cells into the alveolar tissue, leading to damage and apoptosis of alveolar cells [10, 12, 13]. Unfortunately, the incidence of BPD in neonatal intensive care units worldwide have not changed in recent decades and there is a huge need for a new strategy to prevent BPD in neonatal care [14].

Heat shock proteins (HSPs) are produced by cells of virtually all living organisms, from bacteria to humans, to provide protection and facilitate cellular recovery after exposure to damaging stimuli such as heat, toxins, oxidative stress [15, 16]. HSPs stabilize proteins and maintain protein activity to inhibit cell apoptosis and promote cell repair [16–18]. They are categorized into families based on their molecular weight and functional attributes. Among HSPs, the 70 kDa heat shock proteins (Hsp70) are the central hubs of the protein quality control network and collaborate with co-chaperones [19]. Hsp70 interacts with a wide spectrum of molecules and plays a cytoprotective role against various cellular stresses. Under pathophysiological conditions, high expression of Hsp70 interacts at several points with apoptotic signaling pathways, allowing cells to survive lethal injury. Hsp70 also possesses anti-inflammatory properties through multiple mechanisms. The presence of Hsp70 in the culture medium can inactivate dendritic cells and some reports have revealed that Hsp70 can promote the production of regulatory T cells (Tregs), which play a role in the development of tolerance and immunoregulation [20]. In some animal experiments and clinical observations, the expression of HSPs are found in lung tissue cells which imply that Hsp70 may plays an important role in the repair and protection of lung injury [21, 22].

Hsp70 has been used as an *in vivo* systemic intervention in several disease models [23–28]. However, there have been no clinical attempts to use Hsp70 to prevent BPD in premature infants. We hypothesized that Hsp70 could be used to prevent hyperoxia related BPD in the neonatal rat model through its anti-apoptotic and anti-inflammatory effects. Here, the Hsp70s induced by heat stress and intraperitoneal injection of recombinant Hsp70 (rHsp70) were use to explored the potential role of Hsp70 in protecting lungs from hyperoxia-induced bronchopulmonary dysplasia.

## Materials and methods

### Animal models

Timed, pregnant Wistar rats were obtained from BioLASCO (Taipei, Taiwan) and housed at the Hospital Laboratory Animal Center in standard environmental conditions. The animal experiments were approved by the Committee for Laboratory Animal Care and Use at Changhua Christian Hospital and followed the guidelines of the National Animal Research Center. All animals were kept pathogen-free with *ad libitum* access to water and food under a 12 h light/12 h dark cycle. The pups were delivered naturally at term and remained with their maternal rats. Maternal rats were swapped every 24 h between normoxic and hyperoxic groups. Each mother received twelve pups.

### Heat-shock pretreatment

Neonatal pups were randomly assigned by nest to two groups to receive whole-body heat stimulation (41°C for 20 min with a blanket) or no heat stimulation. All newborn rats were then placed under hyperoxic conditions (85% O_2_) with a maternal rat.

### Hsp70 treatment

The pups were treated with 200 μg/kg/day of recombinant Hsp70 or 10 ml/kg of phosphate buffered saline (PBS) only, intraperitoneally for 21 days of hyperoxia exposure. At 3,7,14, and 21 days of age, the pups were sacrificed to obtain lung tissue.

### Western blot analysis of Hsp70

Western blot analysis of Hsp70 expression were performed in murine lung tissues resected at three, seven and fourteen days of age. Proteins were transferred to polyvinylidene difluoride membranes (Bio-Rad Laboratories Inc., CA, USA) and immunoblotted with polyclonal rabbit anti-Hsp70 (Enzo Life Sciences, Inc., NY, USA) or anti-β-actin antibodies (Cell Signaling Technology, Danvers, USA). The blots were then incubated with the appropriate horseradish peroxidase-conjugated secondary antibodies, and the bands were visualized using ImmunoStar (Wako Pure Chemical Industries, Ltd., Osaka, Japan). The amount of protein on the membrane was quantified using a chemiluminescence imaging analyzer (ImageQuant LAS 4000 mini, GE Healthcare, Tokyo, Japan), and the expression levels were recorded as ratios of the level of β-actin.

### Isolation and perfusion of the lungs *in situ*

The rats were anesthetized with an intraperitoneal injection of sodium pentobarbital (40 mg/kg). A vertical incision was made along the midline of the thorax and abdomen. An afferent silicone catheter was inserted into the left atrium. The lungs were perfused with saline at a constant flow rate (2–5 ml/min) for 20 min.

### Tissue preparation and morphological assessment of the lungs

After euthanasia, the right lung was embedded in optimal cutting temperature compound (Tissue-Tek, Sakura, Japan) and frozen for immunohistochemistry. The left lungs were inflated with 10% formaldehyde in 20 cm of H_2_O by clamping the trachea. Sections of formalin-fixed, paraffin-embedded blocks were stained with hematoxylin and eosin (H&E) for morphological evaluation. A vertical line was drawn from the center of the respiratory bronchioles to the nearest pleurum or fibrous septum. The number of alveoli on this line is called the radial alveolar count (RAC). A section was randomly selected from each rat and recorded under a microscope (× 100 magnification) using a charge-coupled device camera system (Olympus DP21; Olympus Co., Tokyo, Japan). Five visual fields were randomly selected from each section to calculate the average number, reflecting the number of alveoli in the end-respiratory unit.

### TUNEL assay

Terminal deoxynucleotidyl-transferase-mediated dUTP nick end labeling (TUNEL) analysis of the lung tissue sections was performed using the In Situ Cell Death Detection Kit operation manual. (Cat. 11684817910, Roche), according to the manufacturer’s protocol.

### Immunohistochemistry staining for the distribution of Hsp70 and caspase-3

Paraffin-embedded tissue sections were deparaffinized with xylene and hydrated using graded percentages of alcohol. The tissues were then rinsed with 0.1 M PBS and deionized water several times. Subsequently, the sections were incubated with a 3% hydrogen peroxide solution in methanol for 15 min to quench endogenous peroxidase activity. The sections were incubated with a serum blocking solution for 1 h at 25°C. The sections were then incubated with anti-Hsp70 and anti-caspase-3 antibodies (Sigma-Aldrich Inc., St. Louis, MO, USA) at a 1:100 dilution in primary antibody dilution buffer for 2 h at room temperature, followed by overnight incubation at 4°C. After rinsing three times with 0.1 M PBS containing 0.5% Triton X-100, the sections were incubated with goat anti-rabbit HRP for 1 h at room temperature in a black box. The sections were then dehydrated using various grades of alcohol, washed, and mounted using anti-fade fluorescent mounting medium (Dako Cytomation, Carpinteria, CA, USA). Images were obtained using a microscope (Olympus BX63; Olympus Co., Tokyo, Japan) equipped.

### Statistical analysis

All data are presented as the mean ± standard deviation. Differences between groups were evaluated using two-way analysis of variance with Tukey’s *post hoc* test for all experiments. Statistical significance was set at p < 0.05. All statistical analyses were performed using GraphPad Prism 6.0 software for Windows (San Diego, California, USA)

## Results

### Hyperoxia exposure resulted in BPD-like lung damage

**Fig 1:**
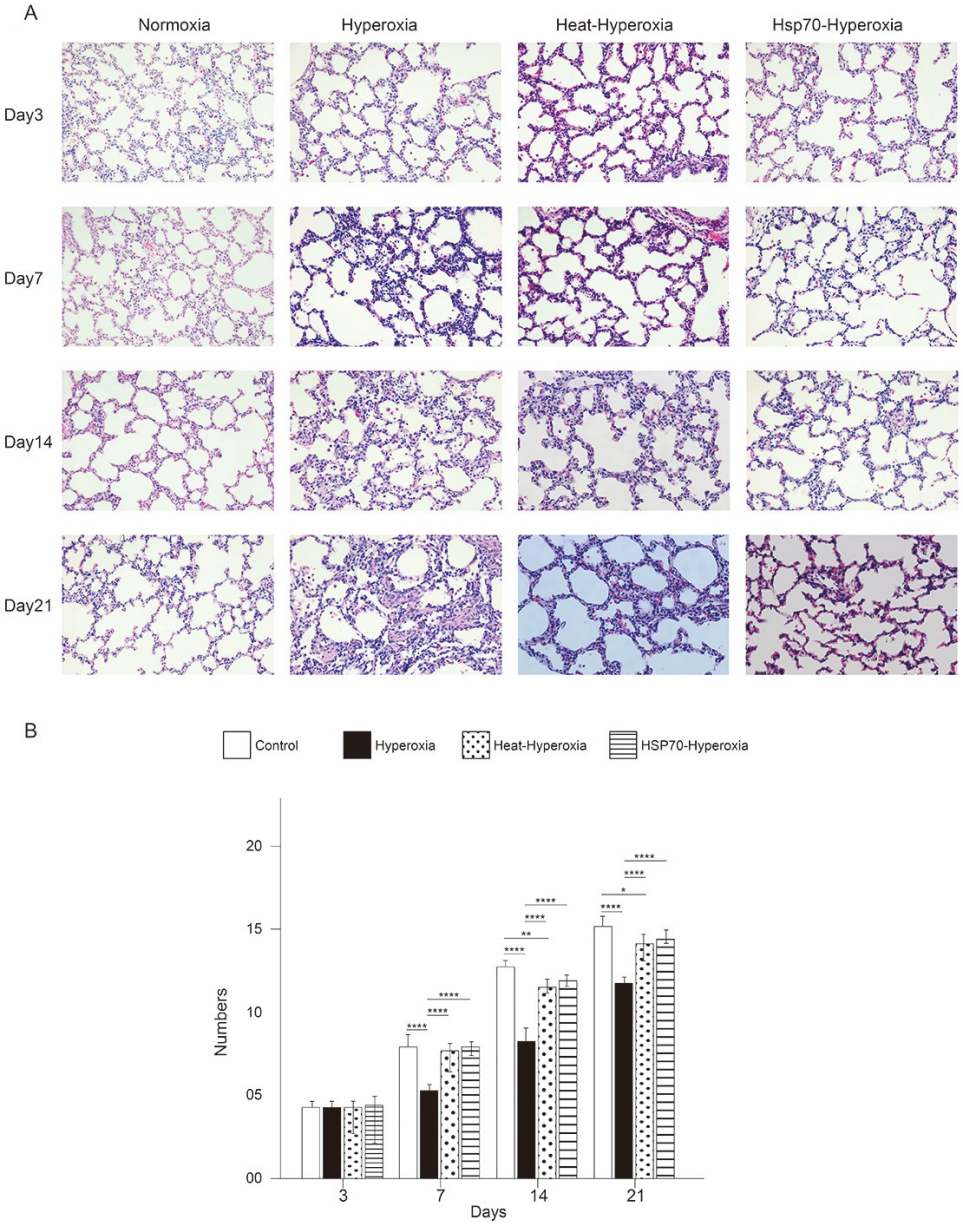
Hyperoxia exposure induces bronchopulmonary dysplasia -like lung injury in neonate rats. Heat shock and exogenous rHsp70 improve hyperoxia lung injury with enhancement of Hsp70. (A) Pathological changes in lung tissue exposed to hyperoxia, heat-hyperoxia, Hsp70-hyperoxia compared to normoxia, 200×) at 3, 7, 14, and 21 days of age. (B) The radial alveolar count (RAC) of the hyperoxia group was substantially lower than that of the treatment groups, *P < 0.05; **P < 0.01; ***P < 0.001, ****P < 0.0001.

Continuous exposure of newborn rats to 85% oxygen for 21 days resulted in marked changes in alveolar development and lung growth. On the third day of hyperoxia exposure, the lung tissue showed slight dilation of the small blood vessels with few foci of hemorrhage and exudation. There were no apparent structural changes after three days of hyperoxia. On the seventh day of hyperoxia exposure, there was a decrease in alveolar numbers with irregular shapes and small collagen deposition. After 14 days of hyperoxia exposure, the alveolar walls were severely damaged with enlarged terminal airways, leading to large and simple acini (Fig 1A). On days 7, 14, and 21, the radial alveolar count (RAC) of the hyperoxia group was substantially lower than that of the treatment group (Fig 1B).

**Fig 2:**
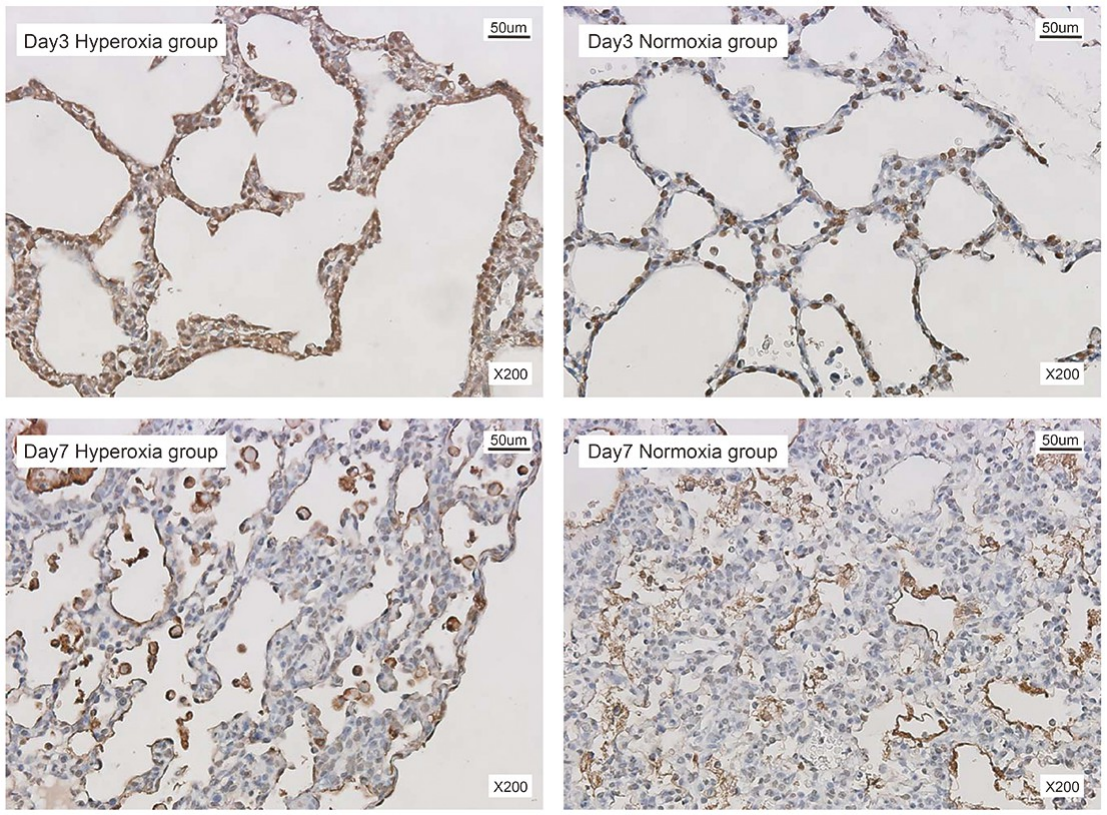
Exposure to hyperoxia induces apoptosis of alveolar cells in the early days. Representative images showing immunohistochemical (IHC) staining of terminal deoxynucleotidyl transferase dUTP nick end labeling (TUNEL) in lung sections in experimental groups. TUNEL-positive cells are indicated by brown while the remaining cells appear cyan with methyl green stain (200 ×).

A mild and progressive inflammatory response was observed in histological sections on the third day of hyperoxia exposure. Inflammatory cells were mainly found in the perivascular and peribronchiolar regions. Initially, neutrophils were the dominant inflammatory cells; then, macrophages gradually became the main inflammatory cells and accumulated in the lungs. In the hyperoxia-exposed group (compared to the normoxia group), alveolar epithelial cell apoptosis occurred on the third day but was weakly expressed on the seventh day of exposure (as indicated by immunofluorescence staining of the TUNEL assay) (Fig 2).

### Heat shock and peritoneal injection of rHsp70 reduced the mortality rates of neonatal rats exposed to hyperoxia

**Fig 3:**
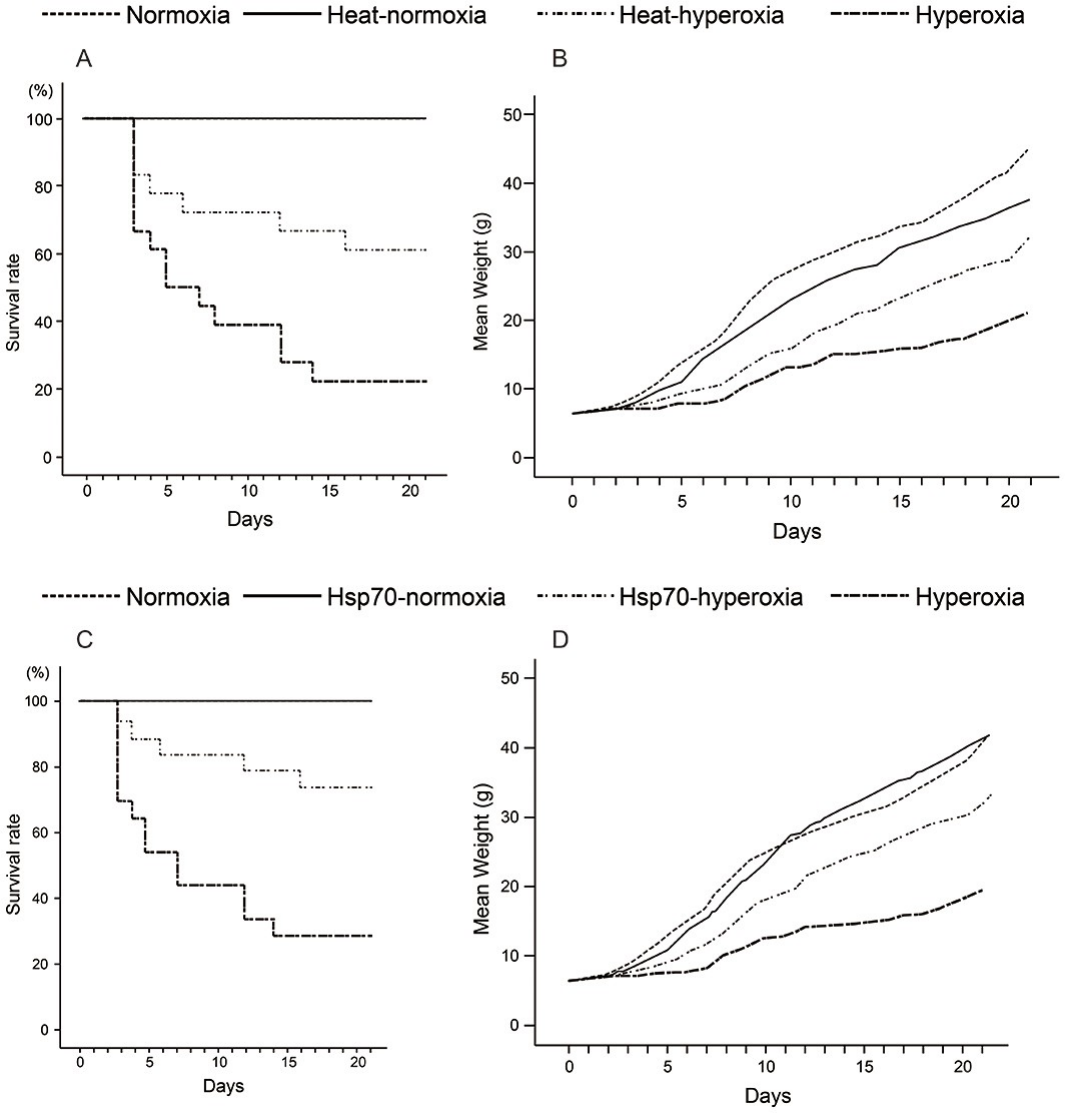
Heat shock and exogenous rHsp70 reduce the mortality and weight change of neonatal rats exposed to hyperoxia. (A) During high concentration oxygen exposure, the survival rate of newborn rats treated with heat shock was higher than that of the hyperoxia group (21% vs 62%, P = 0.018, n = 12). (B) Heat shock pretreatment prevented hyperoxia-induced failure to thrive. (C) During hyperoxia exposure, the survival rate of newborn rats injected with rHsp70 was higher than that of the untreated group (22.2% vs. 73.5%, P = 0.013, n = 12). (D) Treatment with exogenous rHsp70 prevented hyperoxia-induced failure to thrive.

The hyperoxia-exposed neonates began to die on day three. Heat shock significantly increased the survival rate. The mortality rate was reduced from 79% to 38% after heat shock pretreatment (Fig 3A). Furthermore, neonatal rats that received heat shock pretreatment exhibited less weight loss than the hyperoxia group (Fig 3B). To prove that Hsp70 could remediate lung damage, exogenous rHsp70 was used instead of heat shock to treat neonatal rats exposed to hyperoxia. Similar to the effects of heat shock pre-treatment, the survival rate at 21 days increased (22% in the hyperoxia group and 73% in the Hsp70-hyperoxia group) (Fig 3C). As with heat shock, neonate rats receiving rHsp70 injections showed better weight gain than the hyperoxia group (Fig 3D).

### Heat shock and peritoneal injection of rHsp70 attenuated hyperoxia-induced lung injury

**Fig 4:**
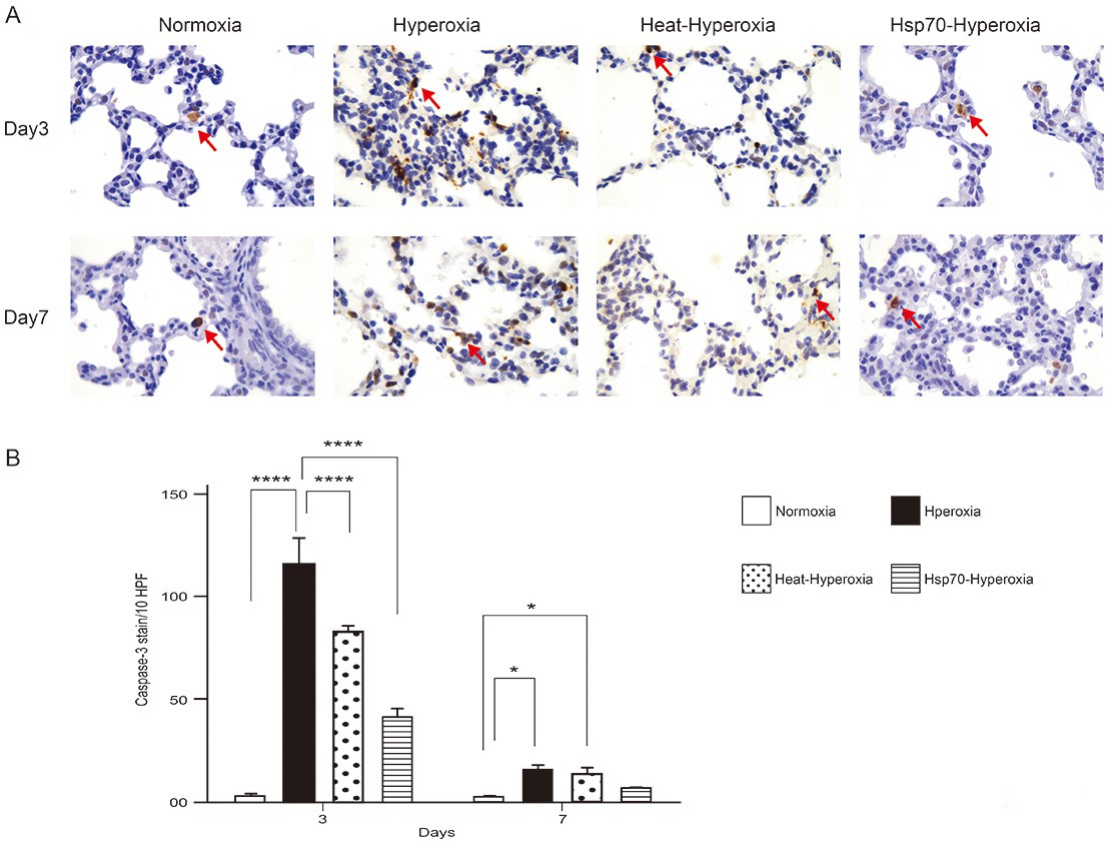
Heat shock and exogenous Hsp70 treatment attenuates hyperoxia-induced alveolar apoptosis. (A) Representative immunohistochemical images for cleaved caspase-3 counterstained with hematoxylin in each group on the third and seventh days of hyperoxia exposure. Red arrows indicate caspase-3-positive cells. Magnification: 630×. Scale bars = 50 mm (B) Quantification of cleaved caspase-3 positive cells in groups. Cleaved caspase-3 was determined by counting the number of red arrows per high-power field in five areas of the lung sections. (400×). The results are presented as mean ± standard deviation (n = 8 in each group). *P < 0.05; **P < 0.01; ***P < 0.001, ****P < 0.0001.

Compared to the hyperoxia-exposed group, the heat-hyperoxia group had a uniform alveolar size, smaller terminal air spaces, and more alveoli on day 7. On the 14th day, the heat-hyperoxia group continued to have better alveolar development, including intact alveolar septa and acinar structures, good alveolar quantities, and a significantly higher RAC (Fig 1A and 1B). The histology of lung tissue on days 3 to 7 of Hsp70-hyperoxia group revealed the same therapeutic change as heat shock. The injection of Hsp70 increased the number of alveoli and maintained their regular size (Fig 1A and 1B). More importantly, heat shock and exogenous Hsp70 treatment significantly reduced alveolar cell apoptosis on the third day after exposure to high oxygen concentrations (Fig 4A and 4B).

### Heat shock and peritoneal injection of rHsp70 both enhanced Hsp70 expression in alveolar epithelial cell

**Fig 5:**
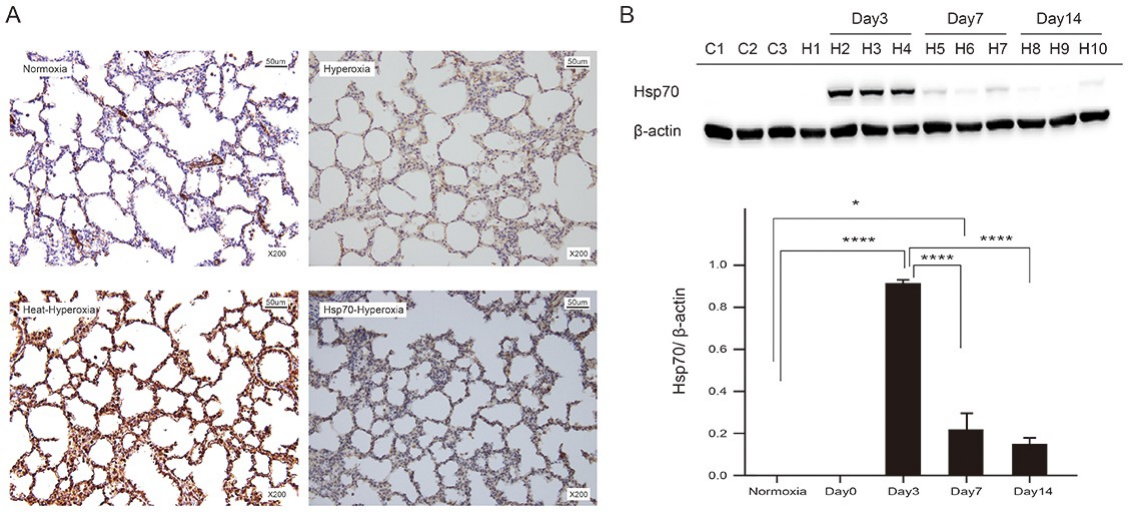
Heat shock pretreatment and peritoneal injection of rHsp70 both enhances Hsp70 expression in the lungs. (A) The panel shows the representative image of Hsp70 expression in the lungs detected by immunohistochemistry (IHC). Strong, positive staining of Hsp70 was evident in the lungs, particularly in the epithelial cells of the pre-heat treatment and exogenous Hsp70 groups compared to the control group (200×; IHC; 3,3’-diaminobenzidine staining). (B) The western blot analysis of Hsp70 expression in the four groups. β-actin was used as a loading control. The results are presented as mean ± standard deviation (n = 8 in each group). *P < 0.05; **P < 0.01; ***P < 0.001, ****P < 0.0001.

Immunofluorescence staining and western blot analysis were performed in the lung tissue to determine the expression of Hsp70 in each group. The results showed that neonatal rats receiving heat shock and exogenous Hsp70 treatment had increased Hsp70 expression in alveolar epithelial cells (Fig 5A). Heat shock pretreatment showed substantially higher levels of Hsp70 in lung tissues, which then gradually decreased (Fig 5B). Hsp70 increased significantly in alveolar epithelial cells on day 3 after heat and then decreased with time. Even on day 14, a slight increase in Hsp70 was observed compared to the normoxia group.

### Heat shock and peritoneal injection of rHsp70 suppressed lung inflammation under hyperoxia

**Fig 6:**
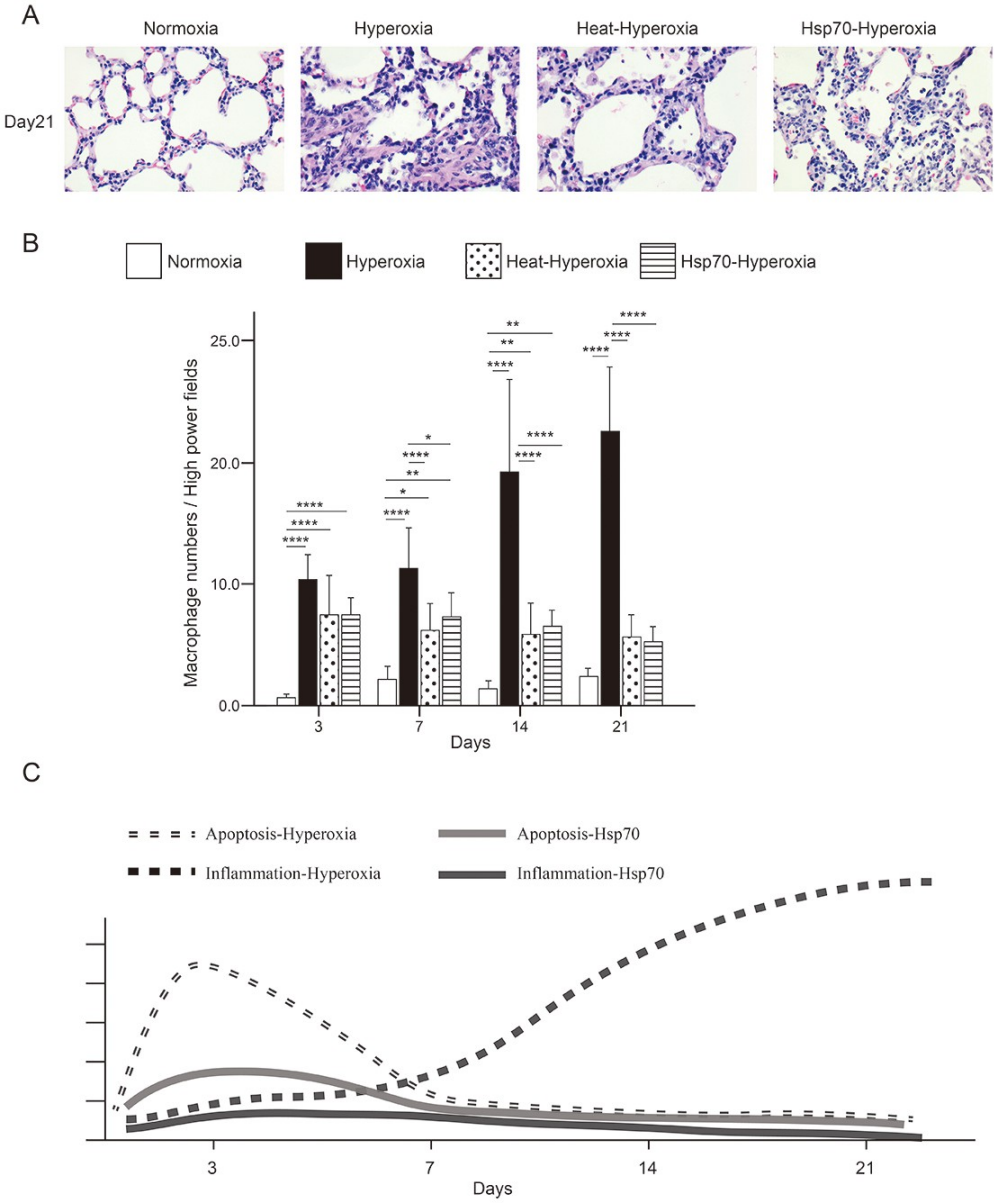
Heat shock and Hsp70 treatment both suppressed lung inflammation under hyperoxia. (A) Accumulation of macrophages in lung tissue of hyperoxia-exposed, heat-hyperoxia, Hsp70-hyperoxia compared to normoxia (400×) at 21 days of age. (B) Infiltrated macrophages in lung homogenates in various groups. Macrophage infiltration was determined by counting the number of macrophages per high-power field in five areas of the lung sections. (400×). (C) Schematic diagram of apoptosis and inflammation under hyperoxia and Hsp70 effect. Data are presented as mean ± standard deviation (n = 8 in each group). *P < 0.05; **P < 0.01; ***P < 0.001.

Quantification of macrophages accumulated in lung sections revealed that heat-hyperoxia and Hsp70-hyperoxia groups showed a significant reduction in lung tissue inflammation (Fig 6A and 6B). Hsp70 injection also reduced the macrophages into the lung tissue (Fig 6A and 6B). Similar to the results of heat shock pretreatment, peritoneal injection of rHsp70 protected against normal alveolar development and decreased lung inflammation under hyperoxic stress.

## Discussion

Hsp70 is closely related to lung biology. In 1998, Wong *et al*. found that hyperoxia-induced cell damage increased Hsp70 expression, which possibly improves hyperoxia-induced lung damage [29]. Additionally, in 2011, Rehan *et al*. evaluated Hsp70 expression in neonate sheep and reported that in lung injuries caused by high airway pressure, the expression of Hsp70 increased, thus improving the inflammatory response [30]. Furthermore, extracellular Hsp70 acts as an immunogen that participates in cross-presentation of MHC-I molecules [31, 32]. Zhang *et al*. demonstrated that extracellular Hsp70 transduces anti-apoptotic and cytoprotective signals through the toll-like receptor 4 and Trif-nuclear factor kappa B pathways, which induces Bcl-2 expression and inhibits caspase-3 activation [33]. Our study is the first one to evaluate the therapeutic potential role of HSP70 in BPD.

Although oxygen therapy is essential for saving the lives of immature infants, prolonged exposure to hyperoxia (even slightly higher oxygen concentrations) can cause lung injury through the production of highly reactive and destructive oxygen radicals, resulting in BPD [11]. Nowadays the pathogenesis of BPD has been gradually elucidated but there have been no breakthroughs in the treatment of BPD. Most of new approaches such as antioxidants, growth factors are not readily applicable to human newborns, owing to safety considerations. Hsp70 is closely related to lung biology, with anti-inflammatory, antioxidant, and anti-apoptotic roles in protecting and repairing lung injury [22, 29]. Since 1992, reports have suggested that an increase in HSP concentration in the lungs can improve mortality associated with acute lung injury in animals [22, 29, 34–40]. Furthermore, highly preserved DNA protein sequences in different species make HSPs prone to fewer immune reactions and have a bigger chance to be used as an exogenous medicine in the neonatal care.

Among the many animal studies investigating hyperoxia and BPD, ours was one of the few that induced exposure to hyperoxia for up to 21 days [41]. Full-term rats are born at the saccular stage of lung development, which corresponds to human lung development between 26 and 28 weeks of gestation. Mouse lung development progresses to the alveolar stage on postnatal day five, which corresponds to human lung development at 32–38 weeks of gestation [42]. Rodents represent the only animal model born at the saccular stage of lung development and are therefore suitable for simulating the hallmark symptoms of neonatal BPD [43]. In our study model, neonatal lungs exposed to hyperoxia exhibited a severely simplified and enlarged alveolar structure, similar to the pathological changes observed in BPD [44–46]. These results demonstrated that the neonate rat model is suitable for BPD researches.

Our results revealed that early damage to the lung by hyperoxia exposure was apoptosis of alveolar rather than inflammation. Alveolar apoptosis occurred mainly on first three days which is similar to previous studies [47, 48]. Both endogenous and exogenous Hsp70 could reduce early apoptosis of alveolar cells under hyperoxia. Although our results and other reports showed hyperoxic stress also induced expression of Hsp70 in alveolar epithelial cells [29, 34], however, it is apparently insufficient to protect the lungs from continuous hyperoxic toxicity.

Macrophages are distributed throughout the lung and can be classified into alveolar macrophages, interstitial macrophages (both are resident macrophages) and recruited macrophages. Moreover, researches had showed that they are heterogenous with functional diversity. For example, alveolar macrophages could produce immunoregulatory cytokines such as TGF-β and IL-10 to keep airway in a state of relative hypo-responsiveness. Severe and persist lung inflammation usually leads to loss of resident macrophages with the replacement, expansion of monocyte-derived macrophages [49, 50]. However, there are studies reported that pulmonary alveolar macrophages are the main contributors to alveolarization arrest in bronchopulmonary dysplasia. Increased expression of pulmonary macrophages was also observed in the lungs of preterm infants who died of BPD [51–53]. The major limitation of our study is lack of flow cytometry analysis to distinguish the different population of lung macrophages or other inflammatory cells. However, from the comparation of different groups, we thought the majority of gradually increased macrophages in hyperoxia group are most likely pro-inflammatory property. Both endogenous and exogenous Hsp70 could suppress macrophage infiltration then results in a reduction in the number of destructed alveoli with fibrosis. In addition to dissect the phenotypes of lung macrophages, to evaluate cytokine expression in different group should be conducted in the future researches.

One interesting thing is that opposed to the continuous injection of rHsp70 for 21 days, the Heat shock induced transient, endogenous Hsp70 for 3 days then gradually decreased after the peak. However, the heat shock group had the same protective effect including anti-inflammation as rHsp70 injection. We do not know the exact reasons of the results but have two possible explanations. The first possibility is that early, sufficient Hsp70 expression could protect the lung organ from hyperoxia damage and let it to develop tolerance to hyperoxic environment, which results in the weak inflammation later even still exposure to hyperoxia. The second possibility is that not only HSP70, the heat shock triggered multiple, unassessed protective mechanisms in our model.

In summary, our results demonstrated that Hsp70 protect the immature lung tissues of neonatal rats from damage caused by hyperoxia through anti-apoptosis in early phase and continues to reduce the inflammatory response (Fig 6C).

## Supporting information

S1 File The data that supports the findings of this study are available in the supporting information of this article. (RAR)
